# Post-flowering biotic and abiotic stresses impact nitrogen use efficiency and seed filling in *Arabidopsis thaliana*

**DOI:** 10.1093/jxb/eraa011

**Published:** 2020-01-13

**Authors:** Anne Marmagne, Sophie Jasinski, Mathilde Fagard, Laurence Bill, Philippe Guerche, Céline Masclaux-Daubresse, Fabien Chardon

**Affiliations:** Institut Jean-Pierre Bourgin, INRAE, AgroParisTech, Université Paris-Saclay,, Versailles, France

**Keywords:** Abiotic stress, biotic stress, drought, harvest index, heat, ^15^N, N recycling, NUE, pathogen attack, senescence

## Abstract

Nitrogen (N) is an essential nutrient that plants require for the synthesis of amino acids, proteins, and many other important metabolites. Plant metabolism and growth are consequently dependent on the amount of N that is assimilated and distributed from source leaves to developing sinks, such as fruits and seeds. The environmental stresses enhanced by climate change deeply influence seed yield and seed composition, and may disturb N use efficiency (NUE) in pants. We aimed to investigate plant responses to extreme climates with regard to NUE, N remobilization efficiency, and seed composition. By studying a collection of Arabidopsis genotypes showing a range of C:N ratios in seeds, we investigated the impact of different post-flowering growth conditions (control, heat, drought, low nitrate availability, induced senescence, and induced plant defense) on seed yield, N allocation in organs, NUE, and N remobilization efficiency. We analysed how post-flowering stresses could change seed filling and showed that post-flowering stresses change both the range of N and C concentrations and the C:N stoichiometry in seeds. Using a new trait, called delta seed composition, we measured the deviation in C:N stoichiometry of each genotype and revealed the genetic determinism of the C:N stoichiometry. Altogether, the results indicate that extreme climate impacts NUE dramatically in plants and generates different bottlenecks in N fluxes during seed filling.

## Teaser

Post-flowering stresses related to climate change impact N management in plants and reveal the crucial role of inflorescence stems in seed filling.

## Introduction

An increasing body of data are revealing the direct and indirect effects on crop production of climate change (Hoegh-Guldberg *et al.*, 2018). The direct factors affecting crop yield are heat stress, drought, and an increase in atmospheric CO_2_ concentrations, while indirect factors include the spread of pests and diseases. The frequency and intensity of extreme events are expected to increase even with relatively small mean climate changes. Understanding the effects of extreme climates on plant yield is critical for policymakers, farmers, and crop breeders to ensure global food security. Massive application of nitrogen (N) fertilizers, mainly in the form of nitrate, has played a key role in improving global food production. Unfortunately, more than half of the N fertilizer is not taken up by crops and is leached in the environment (Lassaletta *et al.*, 2014). Nitrate leaching is a source of nitrous oxide emission that contributes to the excess of long-lived greenhouse gases and consequently participates in global warming (Billen *et al.*, 2013). In this context, there is a pressing need to reduce the use of N fertilizer and to improve N use efficiency (NUE) of crop varieties.

In agriculture, NUE is usually defined as seed yield per unit of N available in the soil, including N fertilizer (Moll *et al.*, 1982). The NUE concept can be split into two aspects: N uptake efficiency (NUpE), which is the ability of plants to take up N from the soil, and N utilization efficiency (NUtE), which is the ability of plants to reduce inorganic forms of N to organic N and to transport N at the whole plant level to produce seeds (Moll *et al.*, 1982). At the plant level, estimation of physiological NUE is complex and can be addressed in different ways. The physiological NUE is usually calculated as the C/N balance in the shoots at harvest, which represents the relationship between the biomass and the N content of the shoots (Good *et al.*, 2004). Another estimation of physiological NUE is determined by the ratio of N harvest index (NHI) to harvest index (HI) (Havé *et al.*, 2017). It estimates the efficiency of a plant in storing N in seeds irrespective of its capacity to produce seeds. Finally, the use of ^15^N tracing, also named ^15^N long-term labeling, allows precise assessment of N dynamics in plants. Quantification of ^15^N in the different plant organs is used to estimate N remobilization efficiency (NRE), which is the capacity of the plant to remobilize the N resources from source organs to new sink organs (Masclaux-Daubresse and Chardon, 2011). Altogether, the different indicators of NUE explain the strategies of N management by plants.

Several scientific papers have reported reduction of yield associated to climate change in crops (Lobell *et al.*, 2011; Leng *et al.*, 2016). Studies on individual abiotic stress confirmed the strong consequences of stress for yield. Indeed, soil dehydration and temperature are important constraints on productivity, seed yield, and seed oil and protein content, especially in rapeseed (Zhao *et al.*, 2017; Hatzig *et al.*, 2018; Sehgal *et al.*, 2018). The reduction of yield might be due to a limitation of floral development caused by water deficit or heat stress in cereals (Barnabás *et al*., 2008). Su *et al.* (2013) revealed that drought stress during seed filling caused an early arrest of floral development and sterility in Arabidopsis. Likewise, Elferjani and Soolanayakanahally (2018) observed that heat stress during the reproductive phase reduced seed yield by 84% and silique number by 43% in rapeseed. Despite this evidence for seed yield susceptibility to environmental stress, the impact on NUE during seed filling still remains poorly described.

The study of the direct and indirect effects of extreme climates on NUE in crops under field conditions is difficult due to the heterogeneity of each experiment and to the complexity of the genotype by environment (G×E) interaction that drives NUE-related traits. The investigation of the G×E interaction on NUE is more feasible in a laboratory with controlled conditions and using model plants, such as Arabidopsis. Indeed, extensive genetic variation for several indicators of NUE has already been demonstrated using natural accessions of Arabidopsis (Diaz *et al.*, 2008; Chardon *et al.*, 2010; Chietera *et al.*, 2018; Meyer *et al.*, 2019). Analysis of this natural variation in NRE allowed us to conclude that the source–sink strength generated by the production of seeds is the main driver of NRE (Masclaux-Daubresse and Chardon, 2011).

In Arabidopsis, some internal and external factors have been reported to modify N management by plants. N availability plays a major role in the mechanism of N uptake and assimilation. In response to external fluctuations of N supply, plants trigger systemic signals for optimizing N uptake and utilization (Xuan *et al.*, 2017). The limitation of nitrate enhances also the remobilization of organic N to support whole plant growth and yield, while reducing N content in seeds (Masclaux-Daubresse and Chardon, 2011). As internal factors, aging and natural senescence of the rosette leaves facilitate N recycling and increases NRE (Diaz *et al.*, 2008; Havé *et al.*, 2017). N nutrition has a significant impact on plant susceptibility to pathogens. Increasing N has been reported to either increase or decrease plant resistance to pathogens depending on the plant–pathogen interaction considered (Mur *et al.*, 2016). Pathogen attacks induce stress effects on plants that can mimic natural senescence (leaf yellowing) and lead to cell death due either to disease development or to active programmed cell death promoted by the hypersensitive response (HR) process. Many *Senescence-Associated Genes* (*SAG*) have been shown to be induced during plant–microbe interactions (Fagard *et al.*, 2014). Among them some genes involved in N remobilization in plants suggest that N recycling is enhanced by some biotic stresses (Pageau *et al.*, 2006; Tavernier *et al.*, 2007).

In addition to the impact on seed yield and NUE of plants, environmental stresses affect seed composition. Several studies in rapeseed reported indeed that heat stress and drought decrease oil content and increase protein content of seeds (Elferjani and Soolanayakanahally, 2018; Zhou *et al.*, 2018). Moreover, together with the reduction of oil content, the composition of individual fatty acids in the seeds is altered under heat stress. Oleic acids (C18:1) decrease whereas linoleic acid (C18:2) and linolenic acid (C18:3) increase in parallel. Interestingly, the protein and lipid contents in oleaginous seeds in Arabidopsis are strongly negatively correlated (Jasinski *et al.*, 2018). Negative correlation between seed protein and lipid content has long been known in rapeseed (Zhao *et al.*, 2006; Jolivet *et al.*, 2013) and sunflower (López Pereira *et al.*, 2000; Li *et al.*, 2017). It is a major obstacle to improving protein content in seeds without modifying the oil yield in plants. The negative relationship between the two contents was revealed by studying several genotypes in field or controlled conditions. The response to environmental stress of this relationship remains unknown.

This study addresses the comparative effects of biotic and abiotic stresses related to extreme climates on NUE and seed filling in Arabidopsis. The study also aims at better understanding the effect of stresses on seed composition with regard to the relationship between C and N in seeds. A collection of Arabidopsis genotypes characterized by different C and N concentrations in their seeds under control conditions were grown under five different stress conditions in addition to the control condition. Stresses applied were: low nitrate availability (low N), heat, drought, dark-induced senescence (dark) and plant infection with an avirulent pathogen (defense). For these plants, 21 traits were measured to evaluate plant growth, seed yield, N partitioning, N remobilization, and seed composition.

## Materials and methods

### Plant material, growth conditions, and stress treatments

Eight recombinant inbred lines (RIL) of Arabidopsis, coming from the *Ct-1*×*Col-0* RIL population, were selected for their different seed N and C concentrations previously observed by Chardon *et al.* (2014). The eight genotypes, named hereafter 7, 63, 83, 134, 359, 397, 401, and 442, showed a range of C/N ratios between 13.3 and 16.1 (see Supplementary Table S1 at *JXB* online). Seeds of the genotypes, previously provided by the Versailles Resource Centre (INRA, Versailles, France, http://publiclines.versailles.inra.fr/), were sown at the same time and plants were grown under the same control growth conditions in order to obtain large quantities of homogeneous seeds, allowing us to use the same batch of seeds in all the following experiments.

Seeds were stratified in tubes containing water in a cold room for 2 d at 4 °C in the dark. After stratification, seeds were directly sown into pots containing compost in a growth chamber or greenhouse, depending on the experiment (Table 1). Plants were placed in the control condition until just after flowering (bolting): in the climatic chamber this was 22/18 °C day/night temperatures, 16/8 h day/night photoperiod, ~180 µmol m^−2^ s^−1^ photon flux, and 60% humidity; in the greenhouse this was 22/18 °C day/night temperatures, 16/8 h day/night photoperiod, natural light with the addition of supplemental light from a high-pressure sodium source, ~440±40 µmol m^−2^ s^−1^ photon flux, and 45–60% humidity. Plants were grown under high N nutrition (10 mM nitrate) in all the control and stress conditions, expect the low nitrate condition, which consisted of growing the plants under low N nutrition (2 mM nitrate) from the beginning. Composition of nutritive solutions is described in Chardon *et al.* (2010). The plants were watered three times per week with the nutritive solutions.

**Table 1.** List of the different treatments included in the experimental design

Experiment	Year	Treatment						Growth condition	Replicates^*a*^
		Control	Drought	Low N	Defense	Dark	Heat		
1	2014	×	×			×		Climatic chamber	3
2	2014	×	×	×	×	×	×	Greenhouse	4
3	2015	×		×				Greenhouse	3
4	2015	×	×		×	×	×	Greenhouse	3
5	2016	×		×		×	×	Greenhouse	4
6	2018	×	×	×	×	×		Greenhouse	4
7	2019	×		×	×			Greenhouse	4
8	2019	×			×		×	Climatic chamber	4

^*a*^ Number of plants per genotype.

Plants were grouped in different sets in which the eight genotypes were represented by three to four plants each (Table 1). At flowering bud stage, a set of plants was kept under the control condition whereas the other sets were transferred for stress treatments. Treatments applied in the different experiments are described in Table 1. For the drought treatment, plants were watered until plant maturity only once a week instead of three times per week. Soil humidity decreased to 30% in the drought condition whereas it remained at 90% in the control condition. For the high temperature treatment, the plants were transferred at bud stage to, and remained until plant maturity in, a climatic room in which temperature was 30 °C and humidity 80%. For dark-induced senescence, tin foil was used to cover the rosette leaves, but not the stem, during 10 d. After 10 d, most of the rosette leaves were white, senescent, and dying. For the induced plant defense treatment, three mature non-senescent leaves of the rosette were inoculated with the avirulent strain *Pseudomonas syringae* pv. tomato DC3000 avrRpm1. Bacteria were grown in King’s B medium with kanamycin (50 μg ml^−1^) at 28 °C. They were re-suspended in 10 mM MgCl_2_ and inoculated into the adaxial side of expanded leaves using a needleless syringe. Inoculum was approximately 10^6^ colony‐forming units per milliliter (OD_600_=0.01).

Plants were harvested at the end of their cycle, when all seeds were mature and the rosettes dry. Samples were separated as (i) rosette, (ii) stem (stem+cauline leaves+empty dry siliques), and (iii) seeds (total seeds). Roots were not harvested, as a large part of them was lost in the dry soil. The dry weight (DW) of rosette, stem, and seeds was determined.

### ^15^N labelling, determination of total nitrogen content and ^15^N abundance

Twenty days after sowing, i.e. 1 week before the flowering bud stage, 1 mL of a 10 mM ^15^NO_3_ 10% enrichment solution was applied near the crown of each plant. To analyse unlabeled samples, a few ^15^NO_3_-free plants were harvested in order to determine the ^15^N natural abundance. After drying and weighing each plant organ, dry material was ground to obtain homogeneous fine powders. Subsamples of 1000–2000 μg were carefully weighed in tin capsules to determine the total C and N percentages (N% and C% as mg (100 mg DW)^−1^) and the ^15^N abundance using a FLASH 2000 Organic Elemental Analyzer (Thermo Fisher Scientific) coupled to a Delta V Advantage isotope ratio mass spectrometer (Thermo Fisher Scientific). The ^15^N abundance in each sample was measured as atom percent and defined as *A*%=100×(^15^N)/(^15^N+ ^14^N). In unlabeled plant controls, *A*%_control_ was *c*. 0.3660. The ^15^N enrichment (*E*%) of the plant material was then calculated as (*A*%_sample_–*A*%_control_). The absolute quantity of N and ^15^N contained in the sample was calculated as QtyN=DW×N% and Qty^15^N= DW×*E*%×N%, respectively. Different parameters used to evaluate HI, NUE, N remobilization, and its components were defined as follows:

$$\begin{array}{l} \begin{array}{c} {\text{HI=DW}}_{\text{seeds}}/(DW_{\text{rosette}}+DW_{stem}+DW_{seeds}),\\ \text{N allocation in rosette}={\text{QtyN}}_{\text{rosette}}/({\text{QtyN}}_{\text{rosette}}+{\text{QtyN}}_{\text{stem}}{\text{+QtyN}}_{\text{seeds}}) \end{array},\\ {\text{N allocation in stem = QtyN}}_{\text{stem}}/({\text{QtyN}}_{\text{rosette}}+{\text{QtyN}}_{\text{stem}}{\text{+QtyN}}_{\text{seeds}}),\\ {\text{N allocation in seeds (NHI) =QtyN}}_{\text{seeds}}/({\text{QtyN}}_{\text{rosette}}+{\text{QtyN}}_{\text{stem}}{\text{+QtyN}}_{\text{seeds}}),\\ {}^{15}{\text{N allocation in rosette = Qty}}^{\text{15}}{\text{N}}_{\text{rosette}}/({\text{Qty}}^{\text{15}}{\text{N}}_{\text{rosette}}+{\text{Qty}}^{\text{15}}{\text{N}}_{\text{stem}}+{\text{Qty}}^{\text{15}}{\text{N}}_{\text{seeds}}),\\ {}^{15}{\text{N allocation in stem = Qty}}^{\text{15}}{\text{N}}_{\text{stem}}/({\text{Qty}}^{\text{15}}{\text{N}}_{\text{rosette}}+{\text{Qty}}^{\text{15}}{\text{N}}_{\text{stem}}+{\text{Qty}}^{\text{15}}{\text{N}}_{\text{seeds}}),\\ {}^{15}{\text{N allocation in seeds = Qty}}^{15}{\text{N}}_{\text{seeds}}/({\text{Qty}}^{\text{15}}{\text{N}}_{\text{rosette}}+{\text{Qty}}^{\text{15}}{\text{N}}_{\text{stem}}+{\text{Qty}}^{\text{15}}{\text{N}}_{\text{seeds}}),\\ {\text{NUtE=(DW}}_{\text{rosette}}{\text{+DW}}_{\text{steme}}{\text{+DW}}_{\text{seeds}}{\text{)/(Qty}}_{\text{rosette}}{\text{+DW}}_{\text{stem}}{\text{+QtyN}}_{\text{seeds}}),\\ \text{NUE = NHI/HI.} \end{array}$$

### Analysis of C:N stoichiometry in seeds

The C:N stoichiometry in seeds was analysed using an orthogonal regression, which minimizes the sum of squared orthogonal distances from the data points to the regression line. C% in seeds was plotted along the *y*-axis and N% in seeds was plotted along the *x*-axis. Slope and intercept of the regression line were computed using the gx.rma function of the R package rgr in R version 3.5.0. Delta seed composition is the orthogonal distance between a data point and the regression line and is determined by the following formula:

DSC=DiffN×DiffC/√(DiffN^2^×DiffC^2^), where DiffN and DiffC are the differences between the data point and the regression line onto N% and C% scales, respectively. DSC is recorded as a positive value or a negative value if the differences, DiffN and DiffC, are either positive or negative.

### Statistical analysis

Three-way ANOVA (R software package) was used to assess the effects of the experiment, genotype, stress condition, and their interactions on the trait variation. Each contrast between the control condition and the other conditions was tested by using estimated marginal means with the function ‘contrast’ of the R package emmeans. Pairwise differences between genotypes were tested by using estimated marginal means with the function CLD of the R package emmeans. Broad-sense heritability (*h*^2^) of trait was estimated from expected mean squares using the following equation:

$$\begin{array}{l} h^{2}=\;\frac{\;\sigma \;Gen}{\begin{array}{c} (\;\sigma \;Gen\;+\frac{\;\sigma \;Gen\times Cond}{n}+\frac{\;\sigma \;Gen\times Exp}{k}\\ +\frac{\;\sigma \;Cond\times Exp}{n\times k}+\frac{\;\sigma \;Gen\times Con\times Exp}{n\times k}+\frac{\sigma E}{n\times k}) \end{array}} \end{array}$$

where σ _Gen_, σ _Gen×Cond_, σ _Gen×Exp_, σ _Cond×Exp_, σ _Gen×Con×Exp_, and σ _E_ are respectively the variances of genetic, genetic×condition interaction, genetic×experiment interaction, condition×experiment interaction and genetic×condition×experiment interactions and residuals; *n* and *k* are the numbers of experiment and condition respectively.

## Results

### Impact of environmental stresses on seed yield, plant growth, and N allocation

**Fig. 1.**
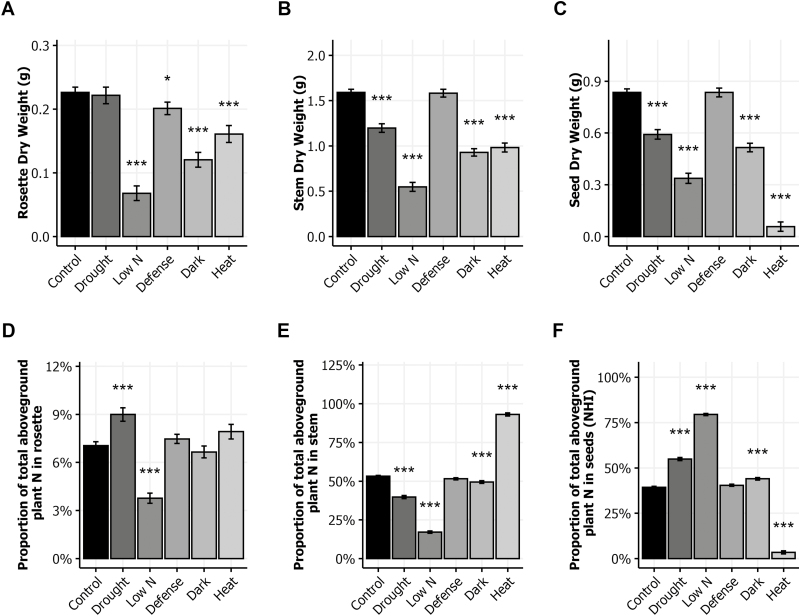
Dry weight and nitrogen allocation in response to environmental stresses. For all the stresses except low N, plants were grown until bolting in the control condition and then transferred to stress conditions (drought, defense, dark, and heat) until seed maturity. The low N condition was applied from sowing. (A–C) The DW of rosette (A), stem (B), and seeds (C) measured at harvest. (D–F) Proportion of total aboveground plant N in rosette (D), stem (E), and seeds (F) of plants grown in the six environments. Means ±SE for the eight genotypes are shown. Asterisks indicate the significance of the difference between the means of the control and stress conditions: *P<0.05, ***P<0.001, *n*≥112.

In order to investigate the response of plant growth, we computed the overall average, the global mean of all the genotypes, for each environment (see Supplementary Fig. S1). We first analysed the overall DW average variations of three plant compartments: rosette, stem, and seeds. In the control condition, DW was on average 0.23, 1.59, and 0.84 g plant^−1^ for rosette, stem, and seeds respectively (Fig. 1A–C). All the stresses limited seed yield or plant growth. The low N condition had the most dramatic effect irrespective of plant organ. The drought condition affected stem and seed DW but had no impact on the rosette. In the heat condition, plants were almost sterile and most of the siliques did not develop fully. The heat stress led to a remarkably low seed DW (0.02 g plant^−1^). When plant defenses were stimulated post-flowering by the avirulent pathogen *Pseudomonas syringae*, there was no modification of stem and seed DW, but surprisingly rosette DW was lower than in control plants. All the treatments limited in different manners the development of the rosette, the inflorescence stems, and the seed yield.

N content analysis of the samples allowed us to quantify the total N content within the aboveground plant (see Supplementary Fig. S2) and then to estimate the N partitioning. Figure 1D–F shows the average N allocation in rosette, stem, and seeds in the six growth conditions. In the control condition, N was allocated at 7%, 53%, and 40% to rosette, stem, and seeds, respectively. All stress conditions significantly modified this distribution, with the exception of the defense condition. The drought condition increased N allocation in the rosette, whereas the low N condition decreased it (Fig. 1D). N allocation to the stem was lower under the drought, dark, and low N conditions, but it was much higher in the heat condition, rising to 95% (Fig. 1E). N allocation to the seeds, the NHI, was much higher in the drought and low N conditions (Fig. 1F). NHI was moderately increased in the dark condition. Heat dramatically decreased NHI, which fell to only 4% of the total N of the plant. The results revealed that even though all the treatments reduced yield or plant growth, their consequences for N partitioning between plant organs were totally different.

### Impact of environmental stresses on harvest index and N use efficiency

**Fig. 2.**
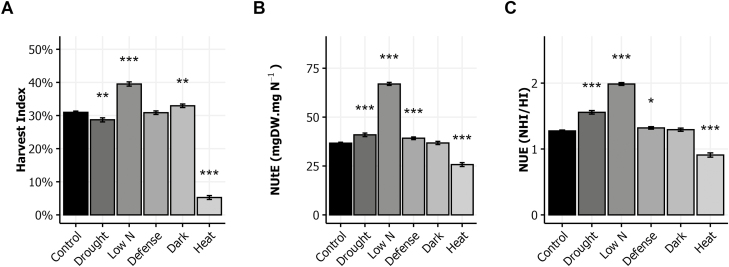
Harvest index and nitrogen use efficiency in response to environmental stresses. Plant material is the same as in Fig. 1. Harvest index (A), nitrogen utilization efficiency (NUtE, B) and nitrogen use efficiency (NUE, C) of plants growing in the six environments. The means ±SE of the eight genotypes are shown. Asterisks indicate the significance of the difference between the means of the control and stress conditions: *P<0.05, **P<0.01, ***P<0.001, *n*≥112.

From the measurements of DW and N allocation, we estimated HI and the two NUE indexes, NUtE and NUE. HI is defined as the ratio of seed DW to aboveground plant DW. In the control condition, HI represented 31% of the aboveground biomass on average (Fig. 2A). It was slightly but significantly lower under drought, higher in the low N and dark conditions, and extremely low in the heat condition. Interestingly, modifications of HI did not follow those of NHI exactly (Figs 1F, 2A). The NUtE was calculated as the ratio of harvested plant DW (mg) to N quantity in harvested plant (mg). It is an estimation of the capacity of the plant to produce biomass based on the quantity of N it can take up. In the control condition, the average of NUtE over all the genotypes was 0.39 (Fig. 2B). In the drought, defense, and low N conditions, NUtE was significantly increased while in the heat condition it was significantly decreased. The NUE index, called hereafter NUE, is the ratio of NHI to HI. NUE reflects the capacity of plants to allocate N to their seeds relative to the allocation of aboveground biomass to the seeds. In the control condition, the average NUE is 1.30 (Fig. 2C). In the drought, low N, and defense conditions, NUE was significantly higher than in the control condition. NUE was lower in the heat condition than in the control condition. These results suggest that the stress treatments have modified the ratio between plant growth and N uptake, as well as the mechanisms involved in the allocation of N resources to the seeds.

### Impact of environmental stresses on N remobilization efficiency

**Fig. 3.**
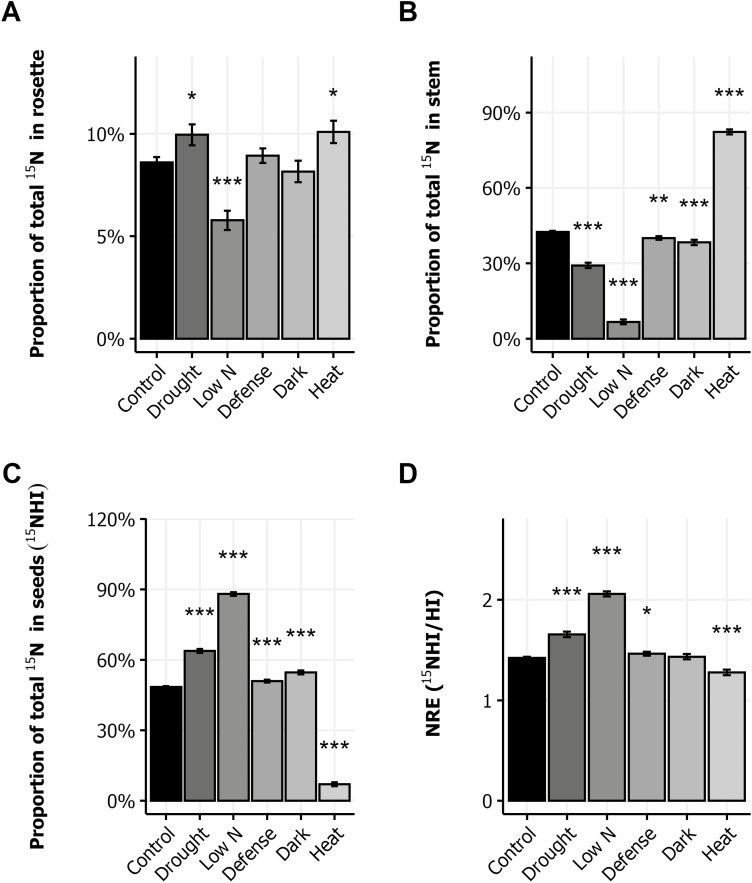
Nitrogen remobilization efficiency in response to environmental stresses. (A–C) Proportion of total ^15^N in rosette (A), stem (B), and seeds (C) in plants grown in the six environments. (D) N remobilization efficiency (NRE) of plants growing in the six environments. The means ±SE of the eight genotypes are shown. Asterisks indicate the significance of the difference between the means of the control and stress conditions: *P<0.05, **P<0.01, ***P<0.001, *n*≥112.

The ^15^N allocation to seeds, hereafter called ^15^NHI according to Masclaux-Daubresse and Chardon (2011), estimates the capacity of plants to remobilize to new sinks the N compounds stored in the rosette during the labeling period. Analysis of ^15^N partitioning to rosette, stem, and seeds indicates where N flux is possibly blocked. In the control condition, 9% of the ^15^N remained in the rosette, 40% was mobilized and stored in the stem, and 51% was mobilized to the seeds (Fig. 3A–C). Compared with the control condition, we observed a slight but significant retention of ^15^N in the rosettes of the drought- and heat-treated plants (Fig. 3A). By contrast, only 6% of the ^15^N remained in the rosettes of the N-limited plants, resulting in an increased N mobilization to the reproductive organs. The ^15^N partitioning in the stem was significantly lower in the drought, dark, defense, and low N conditions compared with the control condition (Fig. 3B). This was due to higher remobilization to the seeds (^15^NHI, Fig. 3C). In the heat condition, due to poor seed production, most of the ^15^N remained allocated in the stem (82% on average). The comparison of ^15^NHI and HI through the NRE index (NRE=^15^NHI/HI) indicates whether or not defects in ^15^N partitioning to seeds are exclusively controlled by sink strength. NRE was on average 1.49 in the control condition (Fig. 3D). It was significantly higher in the drought, low N, and defense conditions, and lower in the heat condition compared with the control condition. We deduced from this that if the sink strength was usually the main driver of N remobilization, some extra processes contributing to N remobilization were enhanced by the drought, defense, and low N treatments.

### Impact of environmental stresses on seed C and N percentages

**Fig. 4.**
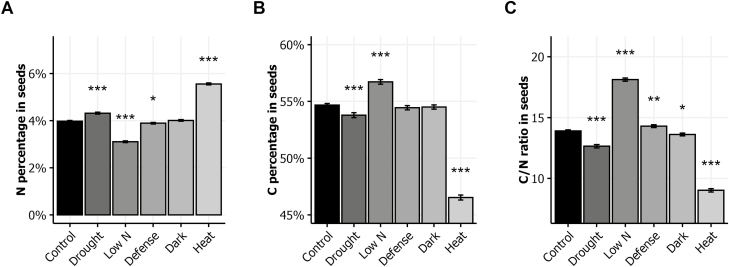
Seed composition in response to environmental stresses. N% (A), C% (B), and C/N ratio (C) in seeds of plants grown in the six environments. The means ±SE of the eight genotypes are shown. Asterisks indicate the significance of the difference between the means in the control and stress conditions: *P<0.05, **P<0.01, ***P<0.001, *n*≥112.

In the control condition, N% was on average 4.0% and C% was 54.7%. The average C/N ratio was then 13.9 (Fig. 4A–C). In the drought and heat conditions, seed N% was significantly higher relative to control, while the low N and defense conditions led to lower seed N% compared with the control condition (Fig. 4A). Contrary to N%, C% was significantly higher in the low N condition and lower in the drought and heat conditions than in the control condition (Fig. 4B). Interestingly, despite the apparent opposite fluctuations of N% and C% in each stress condition, the average of the C/N ratio was modified by all the stress conditions relative to the control condition (Fig. 4C). The C/N ratio was higher in the low N and defense conditions than in the control condition, and lower in the drought, dark, and heat conditions. Plotting the average C% against the average N% for each genotype and each condition showed a strong negative correlation between N% and C% (see Supplementary Fig. S3) in all the six environmental conditions.

**Fig. 5.**
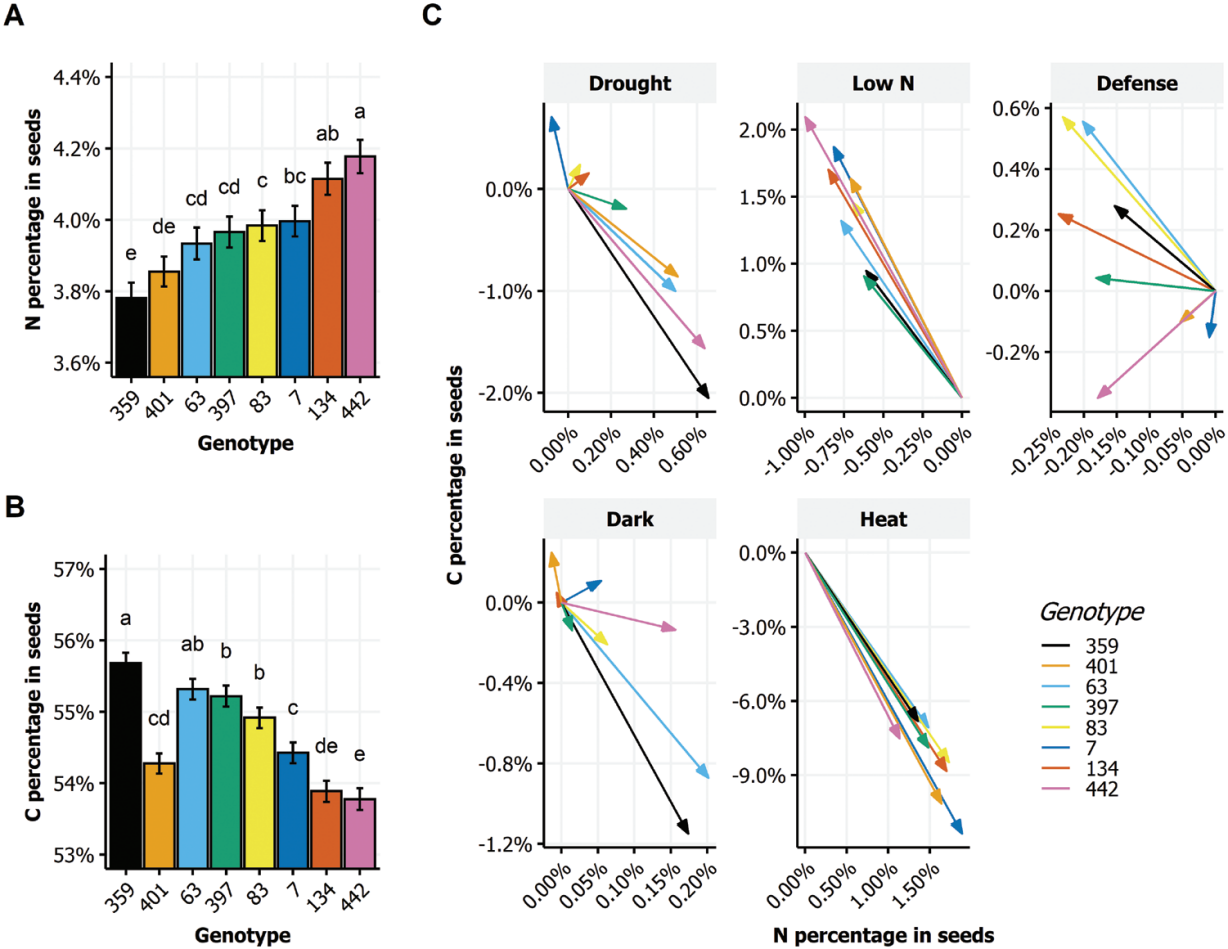
Genotype variability of seed composition among the eight genotypes in the control condition and the relative response to environmental stresses. (A, B) N% (A) and C% (B) in seeds of the eight genotypes (359, 401, 63, 397, 83, 7, 134, and 442) grown in the control condition. Letters indicate statistically significant differences (means ±SE, two-way ANOVA followed by Turkey’s test: *P*<0.05, *n*≥14). (C) Relative response vectors of the eight genotypes to the environmental stresses: drought, low N, defense, dark, and heat. Arrows represent the response vector of seed composition to stress relatively to the seed composition in the control condition.

In the control condition, the eight genotypes were significantly different for both seed N% and C% (Fig. 5A, B). These variations reflect significant differences in the composition of lipids and proteins between genotypes. From the seed composition in the different conditions, we then estimated the relative response of the eight genotypes to the stress conditions using response vectors from the control baseline as shown in Fig. 5C. Vectorization of the stress effect then indicated whether stress-induced variations were going in the same direction for all the genotypes (homogeneous behavior) or were opposite for some (heterogeneous behavior). The strength of the variations was proportional to the length of the vectors. In the low N and heat conditions, variations were strong and homogeneous among the eight genotypes. The drought treatment triggered more heterogeneous effects and caused only moderate variations of seed composition for half of the genotypes. The effects of pathogen infection and dark stress were weak and highly heterogeneous, in direction as well as strength (Fig. 5C).

### Impact of environmental stresses on seed C:N stoichiometry

**Fig. 6.**
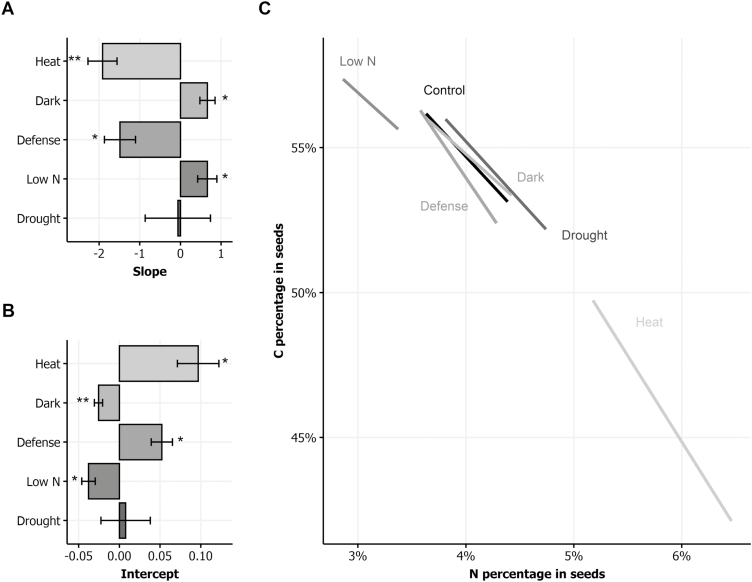
Seed C:N stoichiometry in response to environmental stresses. (A, B) Differences of slope (A) and intercept (B) of the orthogonal regression in the five environmental stress conditions compared with the control condition. Asterisks indicate the significance of the difference between the means in the control and the stress conditions (means ±SD, ANOVA followed by Turkey’s test: *P<0.05, **P<0.01, ***P<0.001, *n*=4–5). (C) Schematic representation of the orthogonal regression of C:N stoichiometry in seeds in the different conditions. The regression parameters in the control condition are the mean of regression parameters estimated in the different experiments. The regression parameters in the stress conditions are the sum of the parameters under the control condition and of the average differences of parameters recorded in the stress conditions. Regression lines are plotted only in the range of seed compositions under the different conditions.

The relative impact of stresses on the seed C:N stoichiometry can be estimated using the slopes and intercepts of the orthogonal regression lines between N% and C% in the six growth conditions. In the control condition, the average (±SD) of the slope considering all the experiments was −4.0 (±0.7) and the average of the intercept was 70.7 (±2.8). The confidence intervals of these two regression parameters indicated that the regression lines slightly changed according to the experiment (see Supplementary Fig. S4). For each experiment, we estimated the changes in slopes and intercepts caused by the stress conditions relatively to the control condition. Figure 6A and B show respectively the relative variations of the slope and intercept in the different stress conditions. Both slope and intercept varied significantly in the low N, dark, defense, and heat conditions. In the low N and dark conditions, the slope and intercept were significantly lower than in the control condition. In the defense and heat conditions, the slope and intercept were significantly higher than in the control condition. Figure 6C illustrates the variation of the regression line in the different conditions and the range of variation of N% and of C% in seeds found in the six conditions. It reveals that treatment could change the seed composition at two levels: (i) in the global range and (ii) in the local C:N stoichiometry. For instance, the seed C:N stoichiometry of plants grown in the low N condition varied from the stoichiometry and the range of variation of seed composition in the control condition (Figs 4, 6). The seed C:N stoichiometry in the dark condition was different from the C:N stoichiometry in the control condition whereas the range of variation of seed composition was similar in the two conditions.

**Fig. 7.**
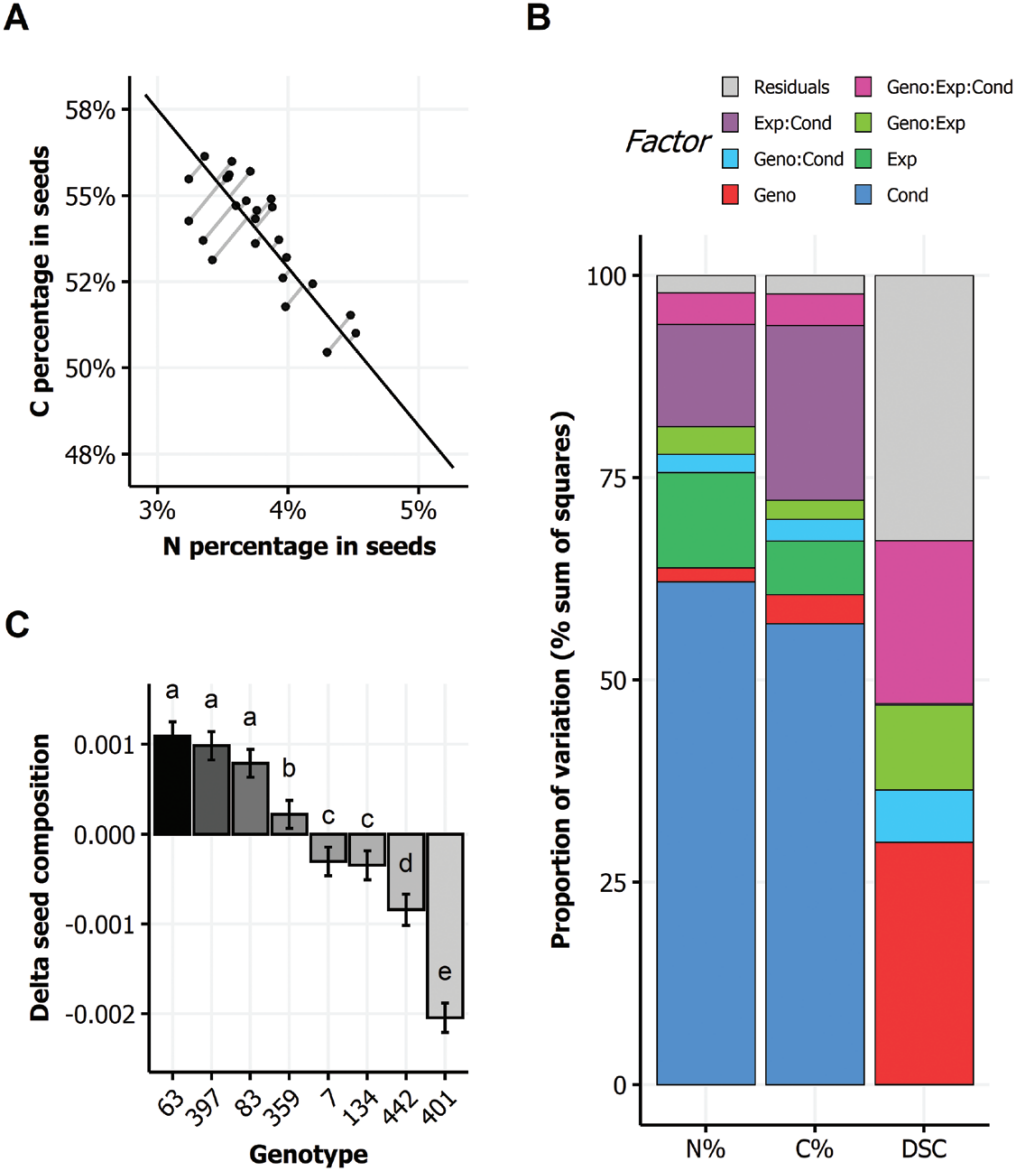
Delta seed composition (DSC). (A) The DSC of plants is the length of the grey segments that join orthogonally each plot to the black regression line; here only data from the control condition of experiment 6 are shown. (B) Proportion of variance explained by factors and interactions in ANOVA shown for N%, C%, and DSC in seeds. Bars show the effects due to genotype (Geno), condition (Cond), and experiment (Exp), and interactions as a percentage of the variation of N%, C%, and DSC in seeds. (C) Genetic variation of DSC among the eight genotypes, irrespective of growth conditions. Letters indicate statistically significant differences (means ±SE, three-way ANOVA followed by Turkey’s test: *P*<0.05, *n*≥14).

For each condition, we considered the orthogonal distance between the observed seed composition and the regression line obtained using all the data of the eight genotypes. This new trait was called delta seed composition (DSC). To figure out better what DSC is, Fig. 7A shows the DSC of plants grown in the control condition and in a single experiment. DSC of each plant can be calculated as in Fig. 7A for all the experiments and growth conditions. Then the DSC variation can be analysed using ANOVA and compared with the ANOVA obtained for seed N% and C% (Fig. 7B). The DSC variation was not explained by the experiment factor nor by the growth condition factor in ANOVA, unlike the seed composition variation. Interestingly, the variation of DSC explained by the genotype factor in ANOVA was much higher than that of seed N% and C%. Accordingly, the broad-sense heritability of DSC was 0.95 whereas it was only 0.44 and 0.63 for N% and C%, respectively. The high heritability of DSC reflects that the position of the genotypes relative to the regression line was remarkably constant and slightly dependent on the environmental condition (Fig. 7C). Regardless of the growth conditions, the genotypes 63, 397, and 83 were usually slightly above the regression line; genotypes 359, 7, and 134 were close to the regression line; and genotypes 442 and 401 were usually slightly below the regression line. Genetic variability of DSC demonstrated that seed C:N stoichiometry is not only determined by the environment but also genetically controlled.

## Discussion

### NUE and NRE plant responses to extreme climates

Our results show that post-flowering drought dramatically reduces stem growth and seed yield, and consequently the HI (Figs 1, 2). Drought also disturbs N allocation in the plant. It increases N allocation to the rosette and seeds but reduces N allocation to the stem (Fig. 1). Similarly, the allocation of ^15^N to the rosette and seeds increases whereas it is reduced in stems, leading to an increase of NRE (Fig. 3). In a previous study, we showed that N remobilization from the rosette to the seeds was mainly driven by the source–sink strength generated by the production of seeds (Masclaux-Daubresse and Chardon, 2011). In the present study, the results suggest that in the drought condition, N remobilization from stem to seeds is strongly increased and compensates the reduction of N remobilization caused by the yield decrease. The post-flowering drought treatment increased moderately seed N% but reduced significantly seed C%, leading to a reduced C/N ratio (Fig. 4). These observed effects of drought on yield and seed composition are in good accordance with previous reports (Bechtold *et al.*, 2010; Elferjani and Soolanayakanahally, 2018). Our study shows the existence of a great natural genetic variability for the plant response to drought (Fig. 5C), as previously reported in Arabidopsis (Bechtold *et al.*, 2010; Easlon *et al.*, 2014; Ferguson *et al.*, 2018). The biggest plants usually show the highest response to drought since they have more stomata, and stomatal transpiration largely controls drought tolerance (Rizhsky *et al.*, 2004). In Arabidopsis, the reduction of the number of stomata is usually associated to lower C assimilation. Because growth rate is much more sensitive to water limitation than photosynthesis, carbohydrates frequently accumulate in drought-stressed plants, showing that growth reduction is not a consequence of carbon deficit (Muller *et al.*, 2011). In our experiments, drought limited C storage in favor of N in seeds (Fig. 4).

The low nitrate availability is well-known to reduce the whole plant weight and seed yield. We found that it also increases HI (Figs 1, 2). The impact of low nitrate fertilization on plant growth and HI has been reported in Arabidopsis and crops (Hirel *et al.*, 2007; Masclaux-Daubresse and Chardon, 2011; Chardon *et al.*, 2012). The low nitrate availability reduces N allocation in rosettes and in stems and favors N allocation to the seeds. This phenomenon reveals higher NRE under low nitrate (Fig. 3), which was already previously demonstrated using a collection of natural accessions (Masclaux-Daubresse and Chardon, 2011). This reflects that plants compensate the low nitrate availability by increasing N recycling from the old organs to the new organs, and in particular from stem to seeds. Despite the higher NRE, seed N% is reduced in plants grown under low nitrate condition (Fig. 4).

The dark-induced senescence of the rosette obtained by covering the leaves during 7 d at the flowering bud stage led to reduced total plant weight and seed yield (Fig. 1). Interestingly, whereas mature rosettes do not form any extra rosette leaves after budding and while mature rosettes were similar before the 7 d stress, after the dark treatment rosettes weighed significantly less than the untreated rosettes. This difference of rosette weight reveals the importance of the post-flowering growth of the rosette, mainly due to the expansion of rosette leaves and the emergence of new axillary leaves at the base of lateral stems. Decreases in stem weight and seed yield come with the reduction of rosette weight under dark treatment (Fig. 1). Similar reductions of seed yield and stem height have been reported in experiments testing the seed production in defoliated plants in Arabidopsis (Akiyama and Ågren, 2012; Easlon *et al.*, 2014; Gnan *et al.*, 2017) and rapeseed (Wang *et al.*, 2016). The sustaining of a minimal seed production highlights the respective contribution of rosette and inflorescence to seed yield. Partial sustaining of seed yield in the dark condition plants might mainly be due to stem and silique photosynthesis (Earley *et al.*, 2009; Gnan *et al.*, 2017). Differences of C assimilation in stems between genotypes may explain the genetic variability of seed composition we observed in response to dark treatment (Fig. 5C). In rapeseed, stems and siliques have also been shown to play a pivotal role in seed filling by contributing together half of C contained within seeds (Brar and Thies, 1977). In a girdling experiment, the removal of the pedicel phloem in rapeseed led to a 10–30% reduction in lipid yield per seed, suggesting that the silique photosynthates were the major contributors to seed weight and oil deposition during maturation (Hua *et al.*, 2012). The dark-induced senescence does not much modify N allocation to rosette whereas it improves N allocation to seeds (Fig. 1). Similarly, it does not change ^15^N allocation to rosette while it reduces ^15^N allocation to stem and increases ^15^N allocation to seeds (Fig. 3). We conclude that the early senescence of the rosette could affect N recycling in stems independently of N availability.

Heat stress remarkably limited the whole plant growth (Fig. 1). Like in the dark condition, the rosette DW at harvest was significantly less for the heat-treated plants than for controls. Even more striking was the reduction of seed yield. Most of the flowers of heat-treated plants did not produce viable siliques. Consequently, HI was dramatically limited (Fig. 2). The source–sink strength exerted by seeds was then very low. Such deleterious effects of heat stress on seed yield have been reported in several Brassicaceae species (Angadi *et al.*, 2000; Morrison and Stewart, 2002; Elferjani and Soolanayakanahally, 2018). Both NUE and NUtE were dramatically lower in our heat-treated plants that were rich in N, suggesting that they are efficient with regard to N uptake. N allocation was also sharply impaired as a result of the very low seed yield and HI (Fig. 1). However, we were surprised to find 95% of the plant N was allocated to stems while N allocation to the rosette was not strongly different from control (Fig. 1). N remobilization to seeds was accordingly extremely low and most of the mobilized N remained in the inflorescence stems (Fig. 3). From these results, we conclude that N remobilization from the rosette to the reproductive organs is not affected by heat stress while N flux from stems into seeds is the limiting step. Whether the low N remobilization was the cause or the consequence of the low seed production remains to be determined. Significant reduction of NRE from stem to grain has been reported under heat stress in wheat (Tahir and Nakata, 2005; Ercoli *et al.*, 2010). In *Brassica junca*, Goel and Singh (2015) reported that heat stress down-regulated the expression of key genes involved in N uptake (nitrate and ammonium transporters) and also in N assimilation (nitrate reductase, nitrite reductase, glutamine synthetase, glutamate synthase, glutamate dehydrogenase, and asparagine synthetase). In Arabidopsis, heat stress promotes the accumulation of some amino acids and of heat shock proteins in the rosette (Kaplan *et al.*, 2004; Rizhsky *et al.*, 2004) suggesting lower N transfer to sinks (Fig. 3). In accordance with the low NUE and NUtE observed, the seeds of the heat-treated plants presented high N% and low C%, and this despite of their low NRE (Fig. 4). This seed composition may have been caused by a high disequilibrium in the ratio of N available in these plants and the few seeds produced. Another cause could be the negative effect of high temperatures on photosynthesis and carbohydrate synthesis. Recently, Zhou *et al.* (2018) specified that high heat stress during the night up-regulates lipases able to degrade storage lipids and fatty-acid desaturating enzymes.

Infection of rosette leaves with the avirulent DC3000 avrRpm1 strain of *Pseudomnas syringae* bacteria activates the plant immune response, which represents a costly energy loss for the plant (Nimchuk *et al.*, 2003; Cui *et al.*, 2005). The hypersensitive-response cell death that is induced strictly limits the pathogen’s growth to the infection site and activates a systemic salicylic acid-mediated response (systemic acquired resistance). We observed that plant infection did not significantly affect plant growth (Fig. 1). The yield of infected plants was the same as that of the control. However, the NUE and NUtE were significantly higher in the infected plants (Fig. 2), suggesting that the amount of N allocated to seeds was higher than that predicted considering the HI. In accordance, NRE was slightly but significantly increased suggesting that N remobilization was induced by the pathogen infection (Fig. 3). This was in good accord with studies that supported that N recycling and mobilization are enhanced by biotic stress (Pageau *et al.*, 2006; Tavernier *et al.*, 2007). The C/N ratio was, however, higher in the seeds of infected plants than in those of the control plants (Fig. 4), due to a decrease of seed N%. Biotic stress may then also affect N uptake in plants. Although the genotypic response to infection was heterogeneous, most of the genotypes responded with a reduction of seed N% (Fig. 5C). Because DC3000 avrRpm1 infection activated systemic acquired resistance, we hypothesize that the observed phenotype at the whole plant level is triggered by the higher salicylic acid levels in these plants. Such a hypothesis is in accordance with previous studies in Arabidopsis and mustard that showed that low salicylic acid levels in plants increase seed yield and plant growth, limit leaf senescence, and improve seed N% (Fariduddin *et al.*, 2003; Nimchuk *et al.*, 2003; Abreu and Munné-Bosch, 2009).

### Environmental stresses affect seed filling and seed C:N stoichiometry

Taking advantage of the eight genotypes chosen on the basis of their C/N ratio in seeds (Chardon *et al.*, 2014), we explored the impact of the environment on the strong negative correlations existing between seed N% and C% (Fig. 4). Our method to provide a quantification of the negative relationship between these traits and to study its responsiveness to the genetic and environmental factors is novel. First, using orthogonal regression, which gives identical roles to seed N% and C%, we investigated the seed C:N stoichiometry in the six environments. Despite small but uncontrolled variation of the regression line in independent experiments in the control condition (see Fig. S4), we found that the environment affected the slope and the intercept of the regression line in addition to the C% and N% (Fig. 6). In the low N and dark conditions, slope and intercept were lower than in the control condition, whereas they were higher for the heat- and pathogen-treated plants. Second, the computing of DSC as a quantitative trait (Fig. 7A) provided a strong heritable trait to consider, by contrast with the N% and C% in seeds (Fig. 7B). We then found some genotypes that were above the regression line (i.e. with positive DSC) irrespective of the environment, and others that were under the regression line (negative DSC; Fig. 7C). The DSC trait may then be used in plant breeding for the improvement of seed composition, in addition to lipid and protein concentrations.

The robust negative relationship between seed N% and C% indicates that the C:N stoichiometry and the protein and oil respective concentrations in seeds are strongly constrained. Two hypotheses are commonly proposed to explain this phenomenon. The first considers the dilution of the protein concentration by the increase of oil concentration, and vice versa. The second considers that C/N balance is controlled by the competition of C and N metabolism for energy. From this point of view, De Vries *et al.* (1974) then proposed a method to estimate the production cost in amount of glucose required to build proteins and oil. In the same vein, other studies predicted the costs of oil, protein, or starch production in wheat, pea, and rapeseed seeds (Morrison *et al.*, 2000; Munier-Jolain and Salon, 2005). While the increase in proteins is predicted to be equivalent to the decrease in oil concentration on an energy basis, an increase of 10% more protein in seeds leads to a cost of +3.16% of energy, mainly due to N uptake and reduction (Munier-Jolain and Salon, 2005). Higher protein content might then imply a supplemental cost that impairs oil production and/or seed yield. In addition to these two hypotheses, we propose a third assumption that the N availability in the plant plays a role in the C:N stoichiometry. The kinetic of N flux and the nature of the N compounds transported in the sieve sap might affect locally the C and N metabolism of siliques. Two pieces of evidence support this hypothesis: (i) the slope of the regression line was lower in the low N and dark conditions that are known to enhance the senescence process (Fig. 6), and (ii) the strong heritability of DSC (Fig. 7C).

## Supplementary data

Supplementary material is available at *JXB* online

Fig. S1. Proportion of variance explained by factors and interactions in ANOVA.

Fig. S2. N quantity in rosette, stem, and seeds of plants grown in the six environments.

Fig. S3. Means of seeds composition of genotypes in the six conditions.

Fig. S4. Relationship between seed N and C of plants grown in the control condition in eight independent experiments.

Table S1. List of selected genotypes and their seed composition.

eraa011_suppl_Supplementary_Table_S1_Figure_S1-S4
