# Supplementary material for: Post-flowering biotic and abiotic stresses impact nitrogen use efficiency and seed filling in Arabidopsis thaliana
Source: J Exp Bot. 2020 Jan 13;71(15):4578–90. doi: 10.1093/jxb/eraa011 (PMC7382380; doi:10.1093/jxb/eraa011)
Supplement: eraa011_suppl_Supplementary_Table_S1_Figure_S1-S4 [file eraa011_suppl_supplementary_table_s1_figure_s1-s4.pdf]

Supplemental Table S1: List of selected genotypes and their seed composition.

Name	N%	C%	C:N ratio
7	3.57	55.45	15.5
63	3.80	55.12	14.5
83	3.57	55.51	15.6
134	3.71	53.82	14.5
359	3.48	55.97	16.1
397	3.63	55.18	15.2
401	3.92	54.52	13.9
442	4.25	53.73	13.3

Data recorded from Chardon *et al.* (2014).

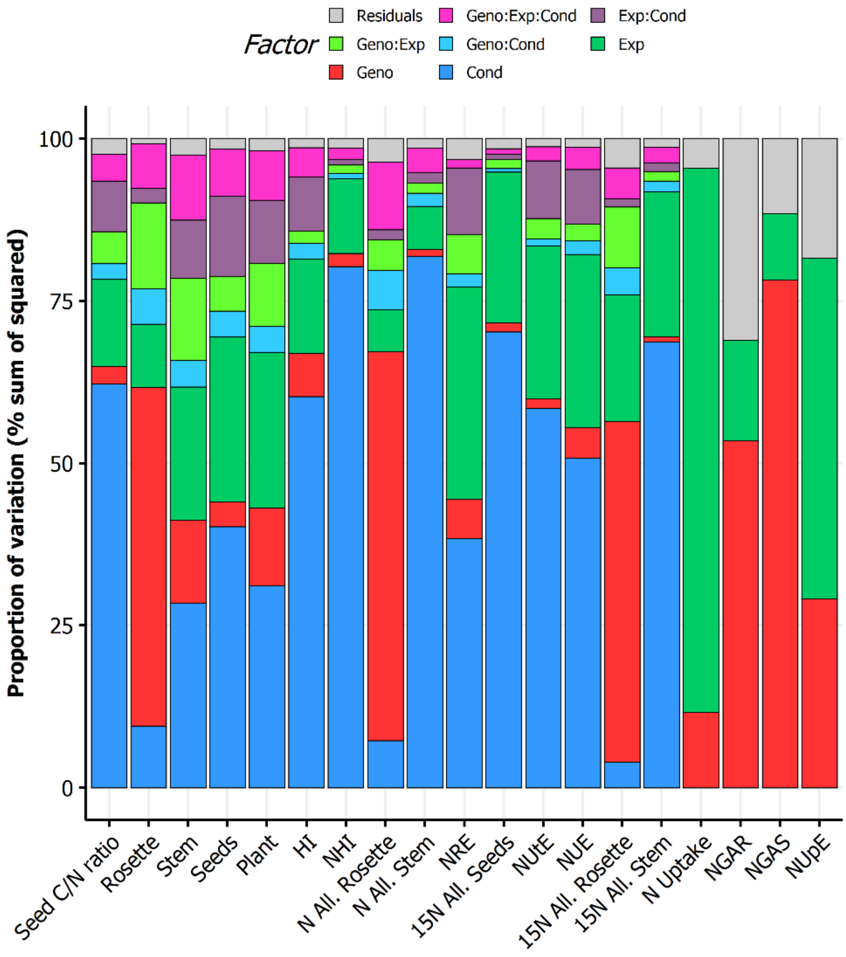

Figure S1: Proportion of variance explained by factors and interactions in ANOVA. Histograms show the effects due to genotype (Geno), condition (Cond) and experiment (Exp), and interactions as a percentage of the variation. Traits analyzed are C/N ratio in seeds (Seed C/N ratio), DW of rosette (Rosette), DW of stem (Stem), DW of seeds (Seeds), DW of the whole plant (Plant), harvest index (HI), N harvest index (NHI Seeds), N allocation to rosette (N All. Rosette), N allocation to stem (N All. Stem), N remobilization efficiency (NRE), ^{15}N allocation to seeds (15N All. Seeds), N utilization efficiency (NutE), N use efficiency (NUE), ^{15}N allocation to rosette (15N All. Rosette), ^{15}N allocation to stem (15N All. Stem), N uptake, N gain allocated to rosette (NGAR), N gain allocated to stem (NGAS), N uptake efficiency (NUpE).

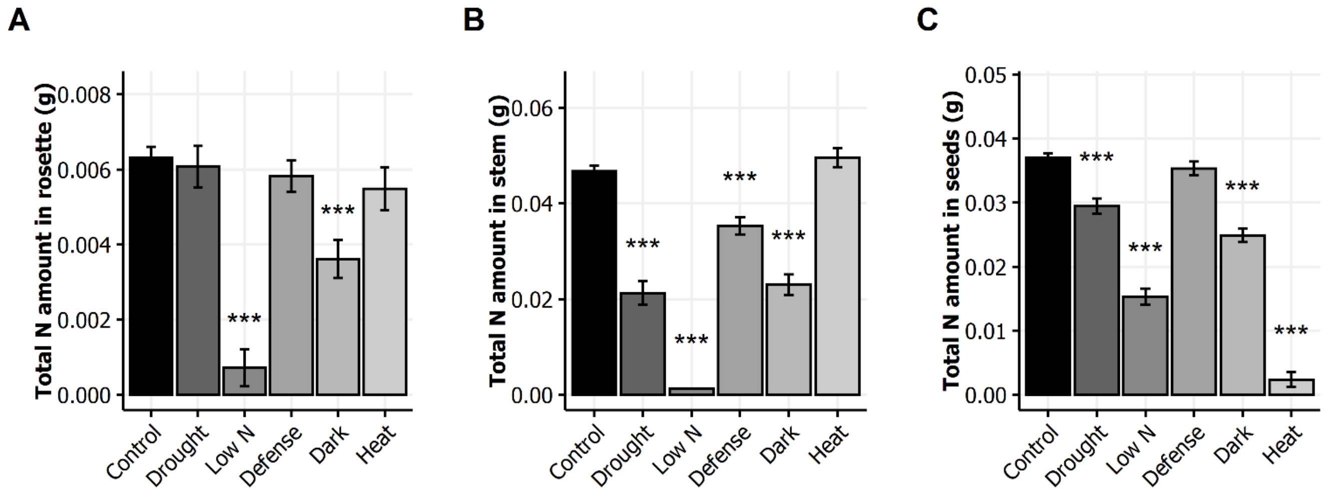

Figure S2: N quantity in rosette, stem and seeds of plants grown in the 6 environments. The mean ± s.e. of the eight genotypes are shown. Stars indicate the significance of the difference between the means of the control and of the stress conditions: * P < 0.05, ** P < 0.01, ***, P < 0.001, n ≥ 112.

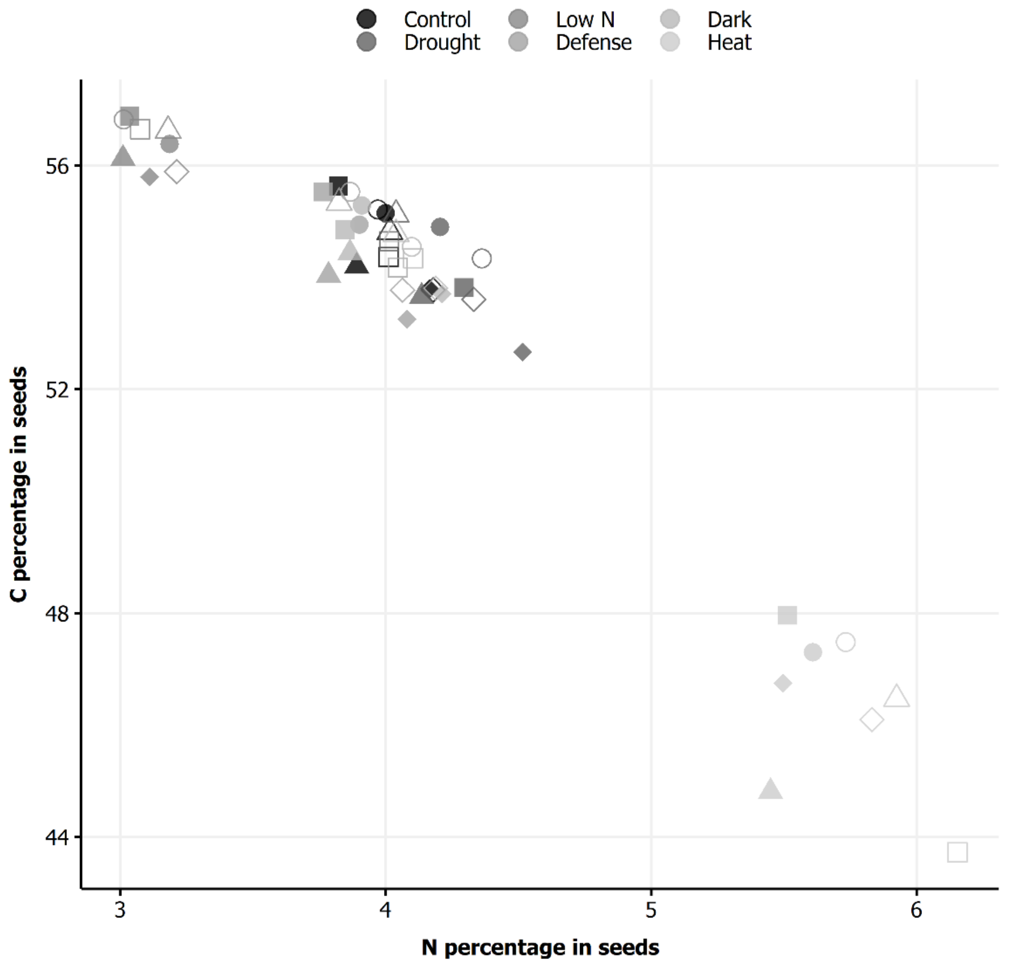

Figure S3: Means of seeds composition of genotypes in the 6 conditions. The plots are colored in a range of grey, from black for the control condition to light grey for the heat condition. Each genotype is shown by the same plot shape in the 6 conditions: 7, square; 63, circle; 83, triangle; 134, filled diamond; 359, filled square; 397, filled circle; 401, filled triangle; 442, filled diamond.

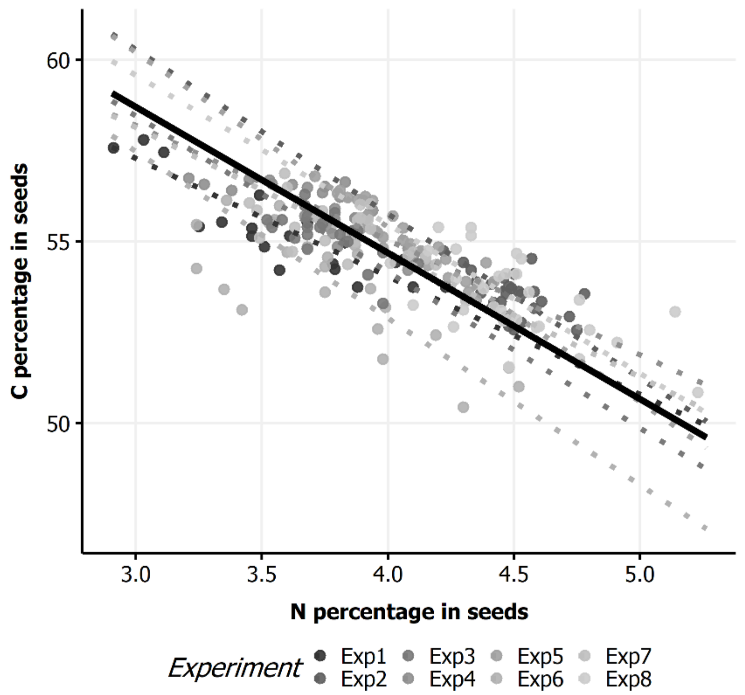

Figure S4: Relationship between seed N% and C% of plants grown in the control condition in 8 independent experiments. Dotted grey lines are the orthogonal regression lines in the 8 experiments. Dark line shows the regression line with average parameters.
